# Medication diaries do not improve outcomes with highly active antiretroviral therapy in Kenyan children: a randomized clinical trial

**DOI:** 10.1186/1758-2652-12-8

**Published:** 2009-06-24

**Authors:** Dalton C Wamalwa, Carey Farquhar, Elizabeth M Obimbo, Sara Selig, Dorothy A Mbori-Ngacha, Barbra A Richardson, Julie Overbaugh, Thaddeus Egondi, Irene Inwani, Grace John-Stewart

**Affiliations:** 1 Department of Paediatrics University of Nairobi, Nairobi, Kenya; 2 Department of Epidemiology, University of Washington, Seattle, USA; 3 Department of Medicine, University of Washington, Seattle, USA; 4 University of Colorado School of Medicine, Denver, USA; 5 Department of Biostatistics, University of Washington, Seattle, USA; 6 Division of Human Biology, Fred Hutchinson Cancer Research Center, Seattle, USA; 7 Kenyatta National Hospital, Nairobi, Kenya

## Abstract

**Background:**

As highly active antiretroviral therapy (HAART) becomes increasingly available to African children, it is important to evaluate simple and feasible methods of improving adherence in order to maximize benefits of therapy.

**Methods:**

HIV-1-infected children initiating World Health Organization non-nucleoside reverse transcriptase-inhibitor-containing first-line HAART regimens were randomized to use medication diaries plus counselling, or counselling only (the control arm of the study). The diaries were completed daily by caregivers of children randomized to the diary and counselling arm for nine months. HIV-1 RNA, CD4+ T cell count, and z-scores for weight-for-age, height-for-age and weight-for-height were measured at a baseline and every three to six months. Self-reported adherence was assessed by questionnaires for nine months.

**Results:**

Ninety HIV-1-infected children initiated HAART, and were followed for a median of 15 months (interquartile range: 2–21). Mean CD4 percentage was 17.2% in the diary arm versus 16.3% in the control arm at six months (p = 0.92), and 17.6% versus 18.9% at 15 months (p = 0.36). Virologic response with HIV-1 RNA of <100 copies/ml at nine months was similar between the two arms (50% for the diary arm and 36% for the control, p = 0.83). The weight-for-age, height-for-age and weight-for-height at three, nine and 15 months after HAART initiation were similar between arms. A trend towards lower self-reported adherence was observed in the diary versus the control arm (85% versus 92%, p = 0.08).

**Conclusion:**

Medication diaries did not improve clinical and virologic response to HAART over a 15-month period. Children had good adherence and clinical response without additional interventions. This suggests that paediatric HAART with conventional counselling can be a successful approach. Further studies on targeted approaches for non-adherent children will be important.

## Introduction

As highly active antiretroviral therapy (HAART) becomes increasingly available to African children, a key priority is to achieve and sustain high levels of adherence in order to prevent the emergence of drug resistance and subsequent treatment failure [1,2]. Current World Health Organization (WHO) recommendations on the use of antiretroviral therapy in resource-limited settings recognize the critical role of adherence in order to achieve clinical and programmatic success [3].

An increasing number of African children are accessing non-nucleoside reverse transcriptase (NNRTI)-based first-line HAART regimens, but second-line regimens remain largely unavailable due to high cost. Although protease inhibitor formulations that do not require refrigeration have recently become available, these are in tablet form and hence difficult to use for very young children [4]. Liquid formulations of protease inhibitor preparations still require refrigeration, which is not widely available in such settings. Excellent adherence is therefore crucial to ensure that children remain on the first-line regimens for as long as possible without developing drug resistance [5-9].

Current strategies advocated by the WHO to achieve high levels of adherence to HAART in resource-limited settings focus on patient education through counselling, enhancing caregiver support by encouraging back up (drug buddies), and disclosure of HIV status. In addition, the use of combined fixed-dose drug formulations to reduce pill burden is advocated [3]. There has been some progress with regards to development of paediatric fixed-drug formulations, and currently, dispersible preparations of stavudine and lamivudine, in combination with nevirapine, are available in a few African countries [10].

Practical aids, including calendars and pill boxes, are also encouraged based on findings from western settings where electronic reminders, pill organizers, and on-line paging systems have been used with variable results [11-16]. These reminder systems have been developed based on the observation that simply forgetting is one of the most common reasons cited for missing doses [16].

The medication diary utilizes the same principle and has been shown to improve adherence to a limited extent in adults [14,17,18]. The medication diary provides patients with a chance to monitor their own adherence during the period between clinic visits by ensuring that a record of daily performance is kept. In addition, unlike electronic reminders, the diary is cheap and requires only literacy on the part of the user, making it attractive for evaluation in resource-limited settings. There is an increasing pool of literate caregivers caring for HIV-1-infected children in Kenya, which makes diary use feasible in this setting [19].

The aim of our study was to assess the effect of medication diaries on adherence and clinical outcomes in HIV-1-infected Kenyan children. Children who met WHO clinical criteria for HAART initiation were randomized either to the use of a medication diary with counselling, or to standard counselling only, and followed prospectively.

## Methods

### Study design and subjects

This was an unblinded randomized trial with use of medication diaries as the intervention, and standardized counselling without diary use as the control. Children recruited into this study were drawn from the paediatric wards and HIV clinic of the Kenyatta National Hospital in Nairobi, Kenya. The hospital is Kenya's main public referral facility and serves as the teaching hospital for the University of Nairobi Medical School. Written informed consent was obtained from all study participants. Verbal assent was obtained from children between ages seven and 12 years. This study received ethical approval from the Institutional Review Boards of the University of Washington and the Kenyatta National Hospital.

To be eligible for inclusion, a child had to be between the ages of 15 months and 12 years, antiretroviral drug-naïve, and have moderate (WHO clinical stage 2 with CD4 <15%) to severe (WHO clinical stage 3 or 4) HIV-1 disease. In addition, literacy on the part of the caregiver and anticipated stay within Nairobi for at least one year post enrolment were required.

Clinical procedures, laboratory monitoring and antiretroviral therapy and follow up were conducted as previously described [20]. Briefly, parents or legal guardians of HIV-1-infected children in the paediatric wards and HIV-1 clinic were invited to the research clinic where further in-depth counselling was done. Those who consented were enrolled into the study.

Clinical evaluation and anthropometry was performed at baseline and monthly thereafter. Baseline laboratory investigations, including full haemogram, T cell lymphocyte subsets (CD4), plasma HIV-1 RNA, and liver function tests were performed; socio-demographic information was obtained by interview. A return appointment was set up a week later for randomization and initiation of antiretroviral therapy. These tests were repeated at three to six monthly intervals during follow up.

### Randomization and diary use

Computer-generated block randomization was used to assign children to the medication diary plus counselling group or to the counselling only group. Caregivers assigned to the diary group were given a simple medication diary at the time of HAART initiation. Instructions on how to complete the diary were given by study physicians and checked by a counsellor.

The diary sheets were in tabular form with the name of each medication appearing in a separate row. The caregiver was asked to place a tick mark in an empty box beside the name of the medication to indicate that the child had been given the medicine. They were asked to not place the tick mark if medicines were not administered. At each appointment, the medication diary was inspected to verify whether it had been completed correctly in the intervening period. Caregivers used the medication diary for the first nine months of the study.

### Antiretroviral therapy and adherence

Children were initiated on first-line antiretroviral drug regimens consisting of two nucleoside reverse transcriptase inhibitors (NRTIs) and one non-nucleoside reverse transcriptase inhibitor (NNRTI), as per Kenya national guidelines [21]. Self-reported adherence was measured at each follow-up visit by asking caregivers to state the number of doses missed, if any, in the previous three days and two weeks. If a caregiver reported missed doses, then the number of missed doses over the entire period since the previous appointment was ascertained.

### Statistical methods

All analyses comparing randomization arms were made using the intention-to-treat approach. We compared continuous variables between the study arms at baseline using the Mann-Whitney U test, and categorical variables using the Pearson chi-square test. Weight-for-age (WAZ), height-for-age (HAZ), and weight-for-height (WHZ) z-scores were computed using EPI Info (Version 3.2, Centers for Disease Control, Atlanta, Georgia).

We compared z-scores, CD4 count and percent, and plasma HIV-1 RNA levels between the two study arms at various follow-up timepoints between three and 15 months post HAART initiation by linear regression. In addition, we compared proportions of children achieving virologic success (<100 copies/ml) between study arms using logistic regression.

Full adherence, defined as no missed doses reported for the three days before the clinic visit, was compared between the two arms with generalized estimating equations to account for multiple visits on the same child. In all comparisons we adjusted for baseline CD4 percentage and parental antiretroviral drug use, which differed significantly between the study arms.

## Results

### Description of study subjects

**Figure 1:**
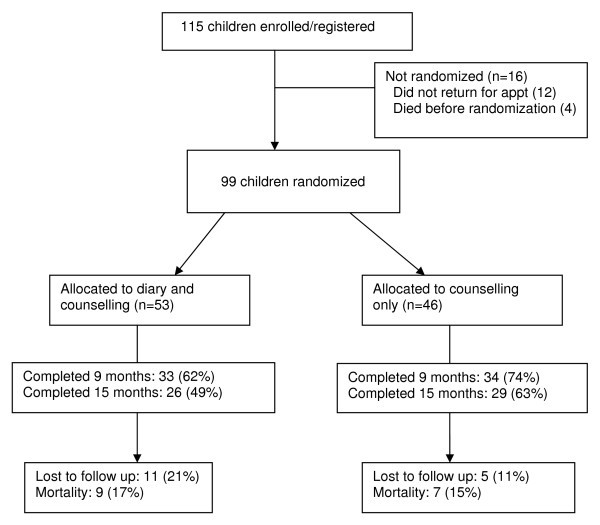
**Flow chart summarizing subject flow**.

A total of 115 children were enrolled between September 2004 and November 2005, of whom 99 were randomized to medication diary plus counselling or counselling alone prior to initiating antiretroviral therapy (Figure 1). Of the 16 enrolled children who were not randomized, four (25%) died before initiation of treatment and 12 (75%) failed to return prior to randomization.

The median age of the 99 children was 4.7 years (interquartile range: 2.3–6.2) and 47 (48%) were male. Of the 99 children eligible for randomization, 53 were assigned to the medication diary group and 46 to the counselling only (control) arm. At baseline, children and their primary caregivers in the two study arms had similar characteristics, except for parental use of HAART and CD4 count and percentage.

Children assigned to the diary arm had higher absolute CD4 count and percentage: median CD4 count of 340 cells/mm^3 ^versus 158 (p = 0.02), and median CD4 percentages of 6.9% versus 5.5% in the diary and control arms respectively (p = 0.05) (Table 1). More parents in the control arm (10, or 22%, of 46 versus one, or 2%, of the 53 in the diary arm) had used HAART at baseline (p = 0.001) (Table 2). Ninety children had any follow-up information available and 67 (68%) children, including 33 (62%) in the diary arm and 34 (74%) in the control arm (p = 0.21), had follow-up information at nine months post HAART initiation. Median length of follow up after initiation of HAART was 15 months (interquartile range: 2–21).

**Table 1:** Characteristics of 99 HIV-1-infected children compared by study arm before initiating HAART

Characteristic	Diary arm (n = 53)		Control arm (n = 46)		p-value
	Median or No*	IQR or %**	Median or No*	IQR or %**	

Age yrs	4.1	2.0, 5.7	5.2	2.7, 6.9	0.15

Males	25*	47**	22*	48**	0.95

Weight-for-age z-score	-2.63	-4.70,1.89	-3.39	-4.80, -1.92	0.52

Height-for-age z-score	-2.16	-3.92, -1.60	-2.27	-3.54, -1.13	0.58

Weight-for-height z-score	-1.37	-2.81, -0.52	-1.81	-3.85, -0.81	0.27

Log_10 _HIV-1 RNA copies/ml	6.1	5.5, 6.5	5.9	5.4, 6.5	0.37

CD4 count/μl	340	138, 663	158	40, 474	0.02

CD4 percent	6.9	3.9, 13.8	5.5	2.0, 9.8	0.05

NNRTI used					

Nevirapine	35*	66**	26*	56**	0.33

Efavirenz	14*	26**	19*	41**	0.12

IQR: interquartile range

NNRTI: Non-nucleoside reverse transcriptase inhibitor

**Table 2:** Caregiver and family characteristics for 99 HIV-1-infected children at baseline

Characteristic	Diary arm n = 53		Control arm n = 46		p-value
	Median or No	IQR or %	Median or No	IQR or %	

Age yrs	29	25, 34	33	27, 37	0.08

Sex, female	45	85	37	80	0.41

Education yrs	10	8, 12	10	8, 12	0.88

Married	31	58	27	59	0.44

Relationship to child:					
Mother Parent	40 46	76 86	28 35	61 76	0.19 0.96

Lost one parent Lost both parents	13 4	25 8	14 2	30 4	0.51 0.50

Shared toilet	44	83	30	65	0.06

Disclosed to relatives	17	32	19	41	0.34

Disclosed to father	28	53	23	50	0.78

Parent used ARV before^a^	1	2	10	22	0.001

Parent tested for HIV^a^	26	49	20	44	0.96

a Data from 19 parents in diary and 24 in control arms respectively

IQR: Interquartile range, ARV: antiretroviral

### Self-reported adherence and tolerance to antiretroviral drugs

A total of 624 questionnaires were filled out for 90 children with follow-up data, and in 553 (89%) questionnaires, no missed doses in the previous three days were reported. In the diary arm, 318 questionnaires were filled out for 47 children, and in 270 (85%), no missed doses were reported. In the control arm, 306 questionnaires were filled out for 43 children, and in 283 (92%), no missed doses were reported.

**Figure 2:**
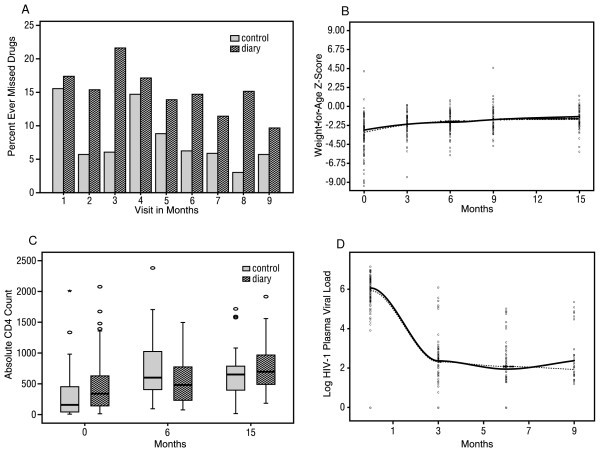
**A – Self reported adherence by study arm; B – Weight-for-age Loess curves by study arm; C – CD4 count by study arm; D – Viral load Loess curves by study arm**.

Using a logistic regression generalized estimating equation model to account for multiple observations per child, there was a trend towards higher self-reported adherence, defined as no missed doses in the previous three days, in the control arm (p = 0.08 adjusted for parental antiretroviral drug use and CD4 percentage at baseline) (Table 3). Eighty six percent of questionnaires completed in the first four months of HAART had no missed doses reported, compared to 91% of those completed after four months (p = 0.05). Figure 2a compares missed doses reported over the previous three days for each study arm over time.

**Table 3:** Outcomes compared between randomization arms adjusted for baseline CD4 percentage and caregiver antiretroviral drug use

Outcome variable	Diary arm		Control		P-value
	Mean or No*	SD or %**	Mean or No	SD or %**	

No missed doses 3-day recall (questionnaire)	270*	85**	283*	92**	0.08^a^
Adherence >95%	35/47	74	38/43	88	0.20

Mortality	9/53	17	7/46	15	0.67

Hospitalization	13/53	25	10/46	22	0.51

Mean CD4 cells/μl					
6 months^b^	585	92	664	105	0.25
15 months^b^	729	95	689	88	0.83

Mean CD4 percent					
6 months^b^	17.2	3.4	16.3	1.6	0.92
15 months^b^	17.6	1.6	18.9	2.0	0.36

Mean HIV-1 RNA log_10 _copies/ml					
3 months (n = 51)	2.69	1.52	2.43	1.17	0.57
6 months (n = 44)	2.27	1.72	2.14	0.94	0.33
9 months (n = 28)	2.77	1.40	2.40	1.27	0.24

VL <100 copies/ml					
3 months	9/27	33	6/24	25	0.61
6 months	10/22	45	11/22	50	0.57
9 months	7/14	50	5/14	36	0.83

Mean WAZ^c^					
3 months (n = 67)	-2.14	0.28	-1.98	0.21	0.99
9 months (n = 62)	-1.36	0.33	-1.55	0.20	0.407
15 months (n = 58)	-1.25	0.23	-1.53	0.25	0.21

Mean HAZ^c^					
3 months (n = 62)	-2.60	0.26	-2.70	0.24	0.62
9 months (n = 61)	-3.73	1.64	-1.84	0.18	0.79
15 months (n = 57)	-1.76	0.25	-3.62	1.82	0.32

Mean WHZ score(SD)^c^					
3 months (n = 55)	-0.29	0.24	-0.68	0.24	0.18
9 months (n = 53)	-0.24	0.30	-0.35	0.25	0.88
15 months (n = 48)	0.21	0.20	-0.18	0.31	0.35

Loss to follow up	12*	23**	6*	13**	0.21

a: GEE (generalized estimating equation) used. b. Data from 20 and 24 children in diary and control respectively c. Numbers for all z-scores month 25 and 28 in diary and control respectively

We defined adherence as being >95% if the caregiver reported never missing a dose or reporting only one missed dose in the past 30 days. In the entire cohort, 73 (81%) of 90 caregivers reported adherence of >95%. In the diary arm, 35 out of 47 (74%) reported adherence of >95% compared to 38 out of 43 (88%) children in the control arm (p = 0.20 adjusted for parental antiretroviral drug use and CD4 percentage at baseline) (Table 3).

Sixteen (18%) of the 90 children – eight in the diary group and eight in the control arm – changed at least one antiretroviral drug due to serious adverse drug effects (eight children), treatment failure (four children), drug interactions with anti-tuberculous drugs (three children), and stavudine being out of stock in the local market (one child). There was no difference between the two arms in frequency of change of antiretroviral drugs regimen (p = 0.79).

### Growth and clinical response

Follow-up z-scores were available at three, nine and 15 months for 25 children in the diary arm, and for 28 in the control arm. After three months of HAART, mean WAZ scores were -2.14 and -1.98 in the diary and control arms respectively (p = 0.99 adjusted for baseline CD4 percentage and parental antiretroviral drug use). The mean WAZ scores after nine months were similar between study arms (Table 3, Figure 2b).

After three months of HAART, the WHZ score was -0.29 in the diary arm versus -0.68 in the control arm (p = 0.18 adjusted for baseline CD4 percentage and parental antiretroviral drug use) (Table 3). As shown in Table 3, the height-for-age z-scores between study arms were not significantly different during the 15 months of follow up. Hospitalization rates were similar between the two arms in the first nine months of treatment (13, or 25%, of 53 in the diary arm versus 10, or 22%, of 46 in the control arm, p = 0.51). There was also no significant difference in mortality between the two arms (nine, or 17%, of 53 in the diary arm versus seven, or 15%, of 46 in the control arm, p = 0.81) (Table 3).

### Immunologic response

CD4 data were available at baseline, six months and 15 months post HAART initiation for 44 children (20 and 24 in the diary and control arms respectively) (Table 3, Figure 2c). The mean CD4 count at six months was 585 and 664 cells/mm^3 ^in the diary and control arms respectively (p = 0.25 adjusted for baseline CD4 percentage and parental antiretroviral use).

After 15 months of HAART, mean CD4 count was similar between the diary and control arms (729 versus 689 cells/mm^3 ^respectively, p = 0.83 adjusted for baseline CD4 percentage and parental antiretroviral drug use). Similarly, there were no significant differences in CD4 percentage between the study arms at six and 15 months (at six months, CD4 percentage was 17.2 versus 16.3, p = 0.92, and at 15 months, it was 17.6 versus 18.9, p = 0.36, in the diary and control arms respectively) (Table 3,).

### Virologic response

Viral load data were available at baseline and at three months for 51 children, including 27 in the diary arm and 24 in the control arm. At six months, viral load data were available for 22 children in each arm, and after nine months of HAART, 14 children in each arm had viral load data. We compared the proportions of children who achieved viral load of <100 copies/ml after three, six and nine months of HAART between the two study arms (Table 3, Figure 2d).

After three months of HAART, nine (33%) of 27 children in the diary arm and six (25%) of 24 children in the control arm achieved viral suppression to levels below 100 copies/ml (p = 0.61 adjusted for baseline CD4 percentage and parental antiretroviral drug use). Six months post HAART initiation, the corresponding figures were 10 (45%) of 22 and 11 (50%) of 22 children respectively (p = 0.57), and after nine months of HAART, the figures were seven (50%) of 14 and five (36%) of 14 children respectively (p = 0.83).

The proportion of children achieving greater than 1.0 log_10 _copies/ml drop in HIV-1 RNA from baseline after three, six and nine months of HAART was similar between children assigned to the medication diary and to counselling alone (Table 3). After controlling for baseline CD4 percentage and parental antiretroviral drug use, the randomization arm was not a predictor of viral suppression below 100 copies/ml at any time point (p = 0.57, p = 0.33 and p = 0.24 for months three, six and nine months respectively).

In the overall cohort children, 15 (39%) of 38 with adherence above 95% achieved viral load of <100 copies/ml after three months, compared to zero out of 13 with adherence of less than 95% (p = 0.007). However, adherence above 95% did not predict viral suppression of <100 copies/ml at six and nine months.

## Discussion

In this study, we report findings of an intervention designed to improve adherence to HAART in children. In our study, use of the a medication diary over the first nine months of antiretroviral therapy did not lead to improved virologic, immunologic, clinical or anthropometric parameters. However, the overall clinical response in this paediatric HIV-1 treatment cohort was good.

Although the randomization groups had similar HIV-1 RNA levels and anthropometric measures at baseline, they differed in some key aspects. The two arms differed significantly with regard to absolute CD4+ T cell count and percentage, with children assigned to the control arm having a lower median absolute CD4 count and percentage. In addition, at baseline, the control arm had a higher proportion of parents who reported prior use of antiretroviral drugs for themselves.

We adjusted for these differences by including baseline CD4 percentage and parental antiretroviral use in all models to address the effect of any residual confounding. However, it is plausible that children in the control arm may have had improved adherence due to these characteristics, thus dampening any potential additional benefit of the medication diary in the intervention arm.

There is evidence that HAART adherence may be better in more symptomatic adults and children due to the perceived benefit of HAART [6,22-24]. The higher frequency of prior parental antiretroviral use in the control arm may reflect a higher proportion of either highly motivated individuals with relative advantages in accessing healthcare, or much sicker parents who were therefore eligible for treatment. Our study was initiated during a period when access to HAART in Kenya was limited almost exclusively to private health facilities.

Another plausible explanation is that adherence was in fact lower in the diary arm, with the diary acting as a barrier through some unforeseen mechanism. The extra time and work it took to record dosing may have made drug administration more cumbersome and time consuming, leading to missed doses because of the perceived lack of time to do it properly. The proportion of questionnaires with no missed doses reported was marginally lower in the diary arm than in the control (85% versus 92%, p = 0.08).

Although self-reported adherence was reasonably high in both study arms (74 and 88% in the diary and control arms respectively, and overall, 81% reporting full adherence), the proportion of children achieving viral suppression below 100 copies/ml over the period of observation was modest, ranging from 33% to 50%. This may be in part due to the high baseline viral load (median 6 log copies/ml) that may require a longer period of time to suppress in children with advanced immunosuppression.

On the other hand, true adherence may be lower than what is reflected by caregiver self-report. Self-reported adherence has been found to overestimate adherence in a number of adult and paediatric studies [25-27]. However, it is reassuring that we observed better early viral response in children with self-reported adherence, >95%, suggesting reliability of self-report in this cohort.

We observed comparable growth, clinical morbidity and viral levels in children in the two trial arms. Given the generally good adherence and clinical responses observed in the absence of interventions, our study provides reassuring evidence that HIV-1-infected children can do well in treatment programmes with conventional counselling. Our study suggests that conventional counselling may be sufficient to promote adherence and improve clinical response and should encourage programmes to more readily provide HAART to children.

Further studies in programme settings will be important to confirm this finding and determine targeted approaches for non-adherent children. Innovative options, such as buddy programmes and enhanced caregiver support, are likely to further improve paediatric care. However, the absence of these interventions programmes should not delay implementing paediatric treatment.

Strengths of our study include: the randomized trial design; the potential feasibility of the intervention; and the multiple outcomes assessed and followed serially for more than a year, including viral and immune markers, adherence, morbidity, survival and growth. The time period of follow up was ideal to test interventions for early adherence that may be critical for setting a good foundation for long-term HAART adherence.

Our cohort experienced significant attrition due to mortality and loss to follow up. This could have the effect of limiting power to detect a difference in outcomes between the study arms at the nine-month and 15-month endpoints, especially if that difference was modest. The diary arm experienced greater loss to follow up than the control arm, which may make it more difficult to detect a difference between the arms at endpoint.

In addition, although the period of our study was sufficient for our aims, ultimately, much longer outcome data up to five years or longer will be important to inform expanding paediatric HIV-1 treatment programmes. It is likely that extended adherence and clinical response are impacted by some factors that differ from early cofactors.

In our study, clinicians were not blinded to the intervention, and diaries were assessed repeatedly during follow up. This may have lead to greater attention being given to the caregivers and children in the diary arm, thereby increasing the likelihood of finding a beneficial effect of the diary. However, we did not find such an effect, which implies that this additional benefit may not have been conferred.

In summary, a simple reminder mechanism, the medication diary used for nine months post HAART initiation, failed to improve clinical and virologic outcomes in HIV-1-infected Kenyan children. Our study illustrates the importance of systematic clinical trials that assess interventions that may otherwise be empirically assumed to be beneficial and initiated without evidence.

The lack of benefit of the diary suggests that different factors than simply forgetting to administer antiretroviral drugs may be important in this setting. Future interventions to improve adherence in HIV-1-infected children in Africa should be designed with the view to address dynamics within the home, including disclosure of the child's HIV-1 status and a family approach, in addition to the premise that caregivers may forget to give medications.

## Competing interests

The authors declare that they have no competing interests.

## Authors' contributions

DW: Lead author, designed and conducted the study led development of the manuscript

CF: Contributed significantly to the design of the study, and manuscript development

EO: Contributed to the care of children in the study and clinical aspects of manuscript development.

SS: Involved in clinical care of children on the study.

DMN: Provided mentorship on design of the study, implementation and manuscript development.

BAR: Provided mentorship on design and led statistical analysis of the data.

JO: Provided mentorship, especially on all lab aspects, and contributed to manuscript development

TE: Provided onsite statistical analysis

II: Contributed to the implementation of the study, care of the children and clinical aspects of manuscript development.

GJS: Provided mentorship in overall design, implementation and epidemiological aspects of manuscript development.
